# The use of text-mining software to facilitate screening of literature on centredness in health care

**DOI:** 10.1186/s13643-023-02242-0

**Published:** 2023-04-29

**Authors:** Emma Forsgren, Sara Wallström, Caroline Feldthusen, Niklas Zechner, Richard Sawatzky, Joakim Öhlén

**Affiliations:** 1 Institute of Health and Care Sciences, Sahlgrenska Academy, University of Gothenburg, Box 457, SE-405 30 Gothenburg, Sweden; 2 University of Gothenburg Centre for Person-Centred Care (GPCC), Sahlgrenska Academy, University of Gothenburg, Gothenburg, Sweden; 3 Department of Forensic Psychiatry, Sahlgrenska University Hospital, Gothenburg, Sweden; 4 Department of Occupational Therapy and Physiotherapy, Sahlgrenska University Hospital, Gothenburg, Sweden; 5 Department of Swedish, Multilingualism, Language Technology, University of Gothenburg, Gothenburg, Sweden; 6 School of Nursing, Trinity Western University, Langley, British Columbia Canada; 7 Centre for Health Evaluation and Outcome Sciences, Providence Health Care, Vancouver, British Columbia Canada; 8 Palliative Centre, Sahlgrenska University Hospital, Gothenburg, Sweden

**Keywords:** Systematic review, Scoping review, Patient-centered care, Person-centred care, Text-mining, EPPI-reviewer, Literature review as topic

## Abstract

Research evidence supporting the implementation of centredness in health care is not easily accessible due to the sheer amount of literature available and the diversity in terminology and conceptualisations used. The use of text-mining functions to semi-automate the process of screening and collating citations for a review is a way of tackling the vast amount of research citations available today. There are several programmes that use text-mining functions to facilitate screening and data extraction for systematic reviews. However, the suitability of these programmes for reviews on broad topics of research, as well as the general uptake by researchers, is unclear. This commentary has a dual aim, which consists in outlining the challenges of screening literature in fields characterised by vague and overlapping conceptualisations, and to exemplify this by exploratory use of text-mining in the context of a scoping review on centredness in health care.

**Supplementary Information:**

The online version contains supplementary material available at 10.1186/s13643-023-02242-0.

## Background

Centredness in health care, i.e. care which takes its starting point in the patient perspective and is co-constructed and managed in partnership between patients and professionals, has been adopted in current health care discourse in Europe [1, 2] and there is an increasing call for its implementation worldwide [3, 4]. The lack of a clear and uniform definition and conceptualisation of centredness in health care is noticeable. However, this has been discussed as a strength, as the contextualisation of a concept is seen as crucial for successful implementation [5]. Nevertheless, the lack of a coherent conceptualisation provides special challenges for literature reviews that explore such a broad topic of research. When decision-makers, practitioners and researchers are unable to review all existing knowledge, there is an obvious risk of misinformation due to a lack of synthesis of all the relevant research.

### Challenges in screening research on centredness in health care

When searching for literature as one of the first steps in conducting a literature review, using a combination of several terms, including index terms and free text words, is most often ideal. This is an important measure to make sure that all relevant literature is retrieved. When focusing on the literature on centredness in health care, this first step presents several challenges. Firstly, various terms are used in connection with centredness, for example, person, patient, client, and family. Secondly, centredness in health care is closely related to and overlaps other fields of research, which themselves involve considerable volumes of publications (e.g. shared decision-making and narrative medicine). Thirdly, only one medical subject heading (MeSH), i.e. “patient-centred care”, exists in relation to the larger field. This MeSH term was introduced to PubMed in 1995 and is defined as: ‘Design of patient care wherein institutional resources and personnel are organized around patients rather than around specialized departments’ [6]. Hence, this term relates mainly to the organisation of care and not its practice and conceptualisation. Also, despite being the only MeSH term for centredness in health care, it is not widely used (only 21,000 hits in the database PubMed) and does not capture the breadth of research literature available.

In order to screen research literature within a reasonable time frame and with the project resources available, the aforementioned challenges can lead to reviews being restrictive in their approach—only using one or a few terms, a limited time frame, or delimiting the screening to a particular population and/or health care context. Even if this is understandable from a pragmatic perspective, the risk is that literature reviews only focus on select parts of the actual field of research and thus do not provide the available evidence.

The difficulty in providing an overall picture of the field becomes clear, for example, when examining one current review, one white paper and an edited volume on person-centred care [1, 7, 8]. Despite overlapping rationales of the publications, there is only minor or no overlap of the included studies. This indicates incompleteness in the syntheses of person-centred research, with the risk of presenting fragmented parts or even only a segment of the larger research field. However, this example is not surprising, since thorough searches in major databases related to centredness in health care end up in more than 90,000 unique citations (which will be further described in detail in our example below). Thus, synthesising this particular field of research involves multi-level challenges, if screening is to be performed manually, which was most likely the case in all of the three review examples described above.

Even if the lack of overlap between publications cannot only be explained by the use of terms, it is a fact that some reviewers choose to use only one term in literature searches, some a couple, while others use several terms. How these choices are made is rarely explained in the literature and is therefore an additional complication. Moreover, according to Hughes and colleagues [9], for example, conceptual differences between constructs are minor and the main difference in terminology depends on the context and patient group in focus. The use of terms does not always correspond with a conceptual basis and several terms are often used in the same publication [10, 11].

### Using text-mining functions in the screening process

A way of tackling the challenge of retrieving an abundance of citations is to use text-mining functions to semi-automate the screening process. Text-mining can be defined as ‘the process of discovering knowledge and structure from unstructured data (i.e. text)’ ([12], p. 2). The use of text-mining within citation screening often entails a classification or prioritisation/ordering of retrieved citations in some way. This process typically involves an iterative approach in which reviewers manually screen titles and abstracts of a set of citations and then use these results to train a statistically predictive classification model to probabilistically identify and order citations by likelihood of relevance.

Text-mining has been used within larger systematic review communities, such as the Cochrane Collaboration, for many years. However, it is likely be increasingly used by smaller working groups (such as ours) as well. Examples of the use of such functions, manually built and tailored for specific review projects, have shown promising results [13, 14]. Such project teams do, however, include expertise in language technology or text-mining.

The development of ‘ready-made’ software available to researchers (not requiring expertise in text-mining) has also rapidly developed during the last couple of years [15]. There are at least fifteen tools incorporating text-mining technologies which are available for abstract and title screening of retrieved citations [16]. The level of uptake for non-experts in text-mining, i.e. researchers, is a question under debate [17]. For current non-users of text-mining, aspects which can hinder uptake are described, for example, the attitude and technological knowledge in a research group (i.e. staff integration), influence from others in the systematic review community (methodological criticism), and possible barriers to organisational and technical integration of software with currently used IT systems. It has been estimated that screening burden can be reduced by between 40 and 90%, i.e. the complete sample of studies needed to be screened to include all relevant records [13, 18]. To reduce screening burden, text-mining and deciding a cut-off or threshold whereby no additional citations require screening are, even if tested for specific as well as broader topics, not widely used due to the risk of lowering the recall [12, 19, 20]. Nevertheless, what these functions can clearly assist with is the earlier identification of the most relevant citations, which can improve the workflow of the complete review [12].

## A case example—screening citations on centredness in health care

Scoping reviews can be described as ‘a preliminary assessment of potential size and scope of available research literature’ ([21], p. 101). This type of review is of particular use when the topic has not yet been extensively reviewed or is of a complex or heterogeneous nature [22]. In our group, we wanted to map research on the topic ‘centredness in health care’. Centredness in health care was defined as care in which (1) the will, needs, and desires of people in need of health care are being elicited and acknowledged and (2) the people in need of health care, health care professionals and other people of importance are working in a collaborative partnership.

A search strategy was developed in several steps using index terms and free text words related to centredness in health care (see Additional files 1 and 2). Relevant records were identified by searching the electronic databases: PubMed, Scopus, PsycINFO, Cumulative Index to Nursing and Allied Health Literature (CINAHL) and Web of Science. Language restriction was English, but no time restriction was applied. To be included in the review, the main aim of the records needed to focus on centredness in health care and the term used needed to be defined and in concordance with our stipulated definition of centredness in health care. The search resulted in the retrieval of 94,236 citations (after removal of duplicates).

In the application of text-mining, we followed the general approach described by Sawatzky et al. [14], which involved first selecting an initial random sample large enough to train a project-tailored classifier model: in our case 5455 records from the database searches. The sample of 5455 records was screened manually (by two reviewers independently against inclusion and exclusion criteria) based on titles and abstracts. Records were classified as “included”, “maybe” or “excluded”. All records labelled “maybe” were screened in full text and then classified as “included” or “excluded”. This specific step was taken to ensure that records labelled as “included” were in fact relevant. The classified records from the manual screening were then used to train a predictive classifier model that was applied to the remaining citations from the database search.

### Manually building a classifier model based on single-word frequencies

We first tried manually building a classifier model. The inspiration for doing this and not employing ready-made software was our experience of successfully building such models in previous work [14], and our lack of knowledge in using ready-made software in the project group at that point in time.

**Fig. 1.**
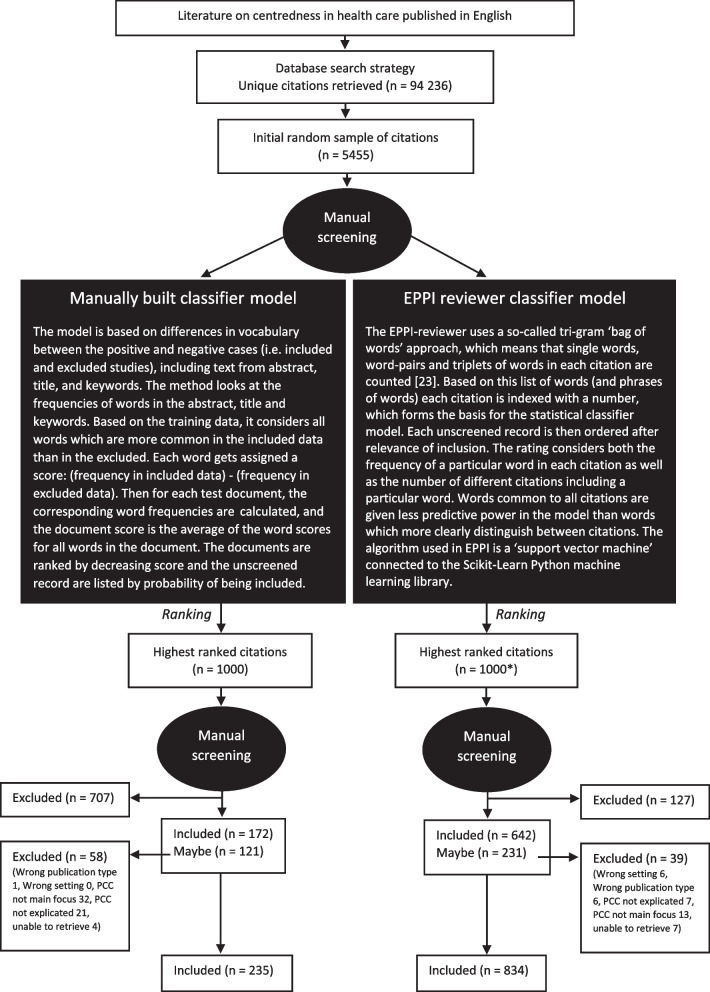
Overview of the two classifier models and the results of the pilot comparison. *Of the 1000 records ranked as most likely to be included in EPPI, 224 records were already included and 79 were records already excluded (using the manually built classifier model), so we did not screen these 303 records again [23]

This manually built model, developed by expert language technologists, was built on single-word frequencies (more information on this model can be found in Fig. 1). However, progressively increasing the accuracy of the manual model proved time-consuming, and it was not possible to overlook the screening burden. This pushed us to consider a ready-made software for screening purposes.

### Building a classifier model based on tri-grams in ready-made software

We decided to test the functionality of the EPPI-reviewer, but this was not straightforward. Although tutorials and support are available for EPPI-reviewer users, the perceived amount of effort needed to use the program was a bit discouraging at the time. Nevertheless, we managed to construct a bespoke classifier model in the program. Like the manual model, this model is built on word frequencies. The difference is that it uses a tri-gram ‘bag of words’ approach, meaning that, in addition to listing single words, word pairs and triplets of words are also recognised and counted for each record. Our model was trained with the results from the random sample screening of the 5455 records, identical to the ones used in the previous step using the manually built classifier model. After this, the EPPI built model was applied to the complete sample of records.

### Pilot comparison

To have a clear rationale for this methodological change in the project, we conducted a pilot comparison between the two project-tailored classifier models, the first one built manually by expert language technologists, and the second one constructed in the program EPPI-reviewer (see Fig. 1).

The 1000 highest-ranking records were retrieved and manually screened for both models. In the manual model, 172 records were included at title and abstract level, 707 excluded and 121 marked as ‘maybes’. When reading these ‘maybes’ in full text, 63 records were included and 58 excluded. As a result, of the 1000 records, 235 were included and 765 excluded, meaning that in total, 23.5% of the sample was included.

In the EPPI model, of the 1000 records screened at title and abstract level, 642 were included, 127 excluded and 231 marked as ‘maybes’. When reading these ‘maybes’ in full text, 192 records were included and 39 excluded. Of the 1000 records, 834 were included and 166 excluded, meaning that in total, 83.4% of the sample was included.

When comparing the two models, of the 235 records which were included using the manually built predictive model for screening, 166 of these were also ranked as highly eligible by the model built in EPPI reviewer. The remaining 69 included records from the manual model were not ranked as highly eligible by EPPI. Nevertheless, the ranking in EPPI resulted in 669 other records being included.

For our purposes, the classifier model built in EPPI-reviewer showed promise in identifying relevant citations earlier in the process, as compared to a manually built classifier. In the manually built model, even if the fraction of positive cases was assumed to be several times higher than for a random sample when ranking citations, it was not expected that the top-ranking citations would ever include more positive (eligible for inclusion) than negative cases (not eligible for inclusion). This was due to the small amount of textual data included in the model (title and abstract) and the skewed distribution (more records labelled as excluded than included in the initial random sample). This expectation was found to be true for the manually built model (based on single-word frequencies) but not for the model in EPPI-reviewer (based on tri-grams) which showed a higher fraction of positive than negative cases.

There is no comparative data available on timeframes or human resources used for screening, but additional rounds of screening from at least two people would be necessary in order for the manual model to identify the same number of included studies as the model built in EPPI-reviewer. Additionally, no formal analysis of the accuracy of the two models was performed.

## Conclusions

The problem of delimiting database searches will not diminish in the future—rather, the opposite is more likely, as the overall number of research publications will increase. Further, new terms and combinations of already implemented terms in connection to centredness in health care might be used.

As Park and Thomas discuss, it is important to consider the specific functions required for a particular review [15]. However, the selection of suitable text-mining functions, as well as their precision for a specific review project, are challenging for a lay text-mining user (an ordinary researcher).

In this commentary, we have discussed challenges associated with screening literature in a field of research with diffuse conceptual boundaries and used an example of our own journey in testing text-mining functions with literature on centredness in health care. The use of ready-made software text-mining functions, such as the ones used in EPPI-reviewer, seems truly promising for large scoping reviews such as ours on topics with diffuse conceptual barriers and large amounts of citations due to systematic database searches.

## Supplementary Information

**Additional file 1.** Search terms.

**Additional file 2.** Search syntax.

## Data Availability

The datasets used and/or analysed during the current study are available from the corresponding author on reasonable request.
