# Supplementary material for: The use of text-mining software to facilitate screening of literature on centredness in health care
Source: Syst Rev. 2023 Apr 29;12:73. doi: 10.1186/s13643-023-02242-0 (PMC10148558; doi:10.1186/s13643-023-02242-0)
Supplement: Supplementary file 1 — Additional file 1. Search terms. [file 13643_2023_2242_MOESM1_ESM.docx]

Additional file 1. Search terms

| Free text words included in searches (all variations in spelling included) | | | Mesh term |
| --- | --- | --- | --- |
| 1. | Person centredness | Person-centred care | Patient-Centered Care |
| 2. | Patient centredness | Patient-centred care |  |
| 3. | Client centredness | Client-centred care |  |
| 4. | Relationship centredness | Relationship-centred care |  |
| 5. | Women centredness | Women-centred care |  |
| 6. | Woman centredness | Woman-centred care |  |
| 7. | Family centredness | Family-centred care |  |
| 8. | Child centredness | Child-centred care |  |
| 9. | People centredness | People-centred care |  |
