# Supplementary material for: The use of text-mining software to facilitate screening of literature on centredness in health care
Source: Syst Rev. 2023 Apr 29;12:73. doi: 10.1186/s13643-023-02242-0 (PMC10148558; doi:10.1186/s13643-023-02242-0)
Supplement: Supplementary file 2 — Additional file 2. Search syntax. [file 13643_2023_2242_MOESM2_ESM.docx]

Additional file 2. Search syntax

| Complete search syntax in Pubmed |
| --- |
| ((“personcent*”[tiab]) OR (“person-cent*”[tiab]) OR (“person cent*”[tiab]) OR (“patientcent*”[tiab]) OR (“patient-cent*”[tiab]) OR (“patient cent*”[tiab]) OR (“Patient-Centered Care”[Mesh]) OR (“clientcent*”[tiab]) OR (“client-cent*”[tiab]) OR (“client cent*”[tiab]) OR (“relationshipcent*”[tiab]) OR (“relationship-cent*”[tiab]) OR (“relationship cent*”[tiab]) OR ("womencent*"[tiab]) OR ("women-cent*"[tiab]) OR ("women cent*"[tiab]) OR ("womancent*"[tiab]) OR ("woman-cent*"[tiab]) OR ("woman cent*"[tiab]) OR ("familycent*"[tiab]) OR ("family-cent*"[tiab]) OR ("family cent*"[tiab]) OR (“childcent*”[tiab]) OR (“child-cent*”[tiab]) OR (“child cent*”[tiab]) OR (“peoplecent*”[tiab]) OR (“people-cent*”[tiab]) OR (“people cent*”[tiab])) |
